# Disparities in Colorectal Cancer Mortality and Survival Trends Among Hispanics Living in Puerto Rico (2000–2021): A Comparison Between Early-Onset and Average-Onset Disease

**DOI:** 10.3390/life15111742

**Published:** 2025-11-13

**Authors:** Camille Montalvo-Pacheco, Carlos R. Torres-Cintrón, Marilyn Moró-Carrión, Hilmaris Centeno-Girona, Luis D. Borrero-García, María González-Pons

**Affiliations:** 1 Division of Clinical and Translational Cancer Research, University of Puerto Rico Comprehensive Cancer Center, San Juan 00921, Puerto Rico; 2 Puerto Rico Central Cancer Registry, San Juan 00921, Puerto Rico

**Keywords:** early-onset colorectal cancer, colorectal cancer mortality, colorectal cancer survival, disparities, racial and ethnic, Hispanic

## Abstract

Colorectal cancer (CRC) is the leading cause of cancer-related death in Puerto Rico, a U.S. territory with noted disparities in CRC incidence, particularly among those with early-onset disease (EOCRC). Although EOCRC incidence has been consistently increasing in the U.S. mainland, and a disparate burden has been reported among Hispanics, EOCRC mortality and survival are yet to be assessed among Hispanics living in Puerto Rico (PRH). In this study, we analyzed EOCRC mortality and survival trends in PRH and compared these to those of other U.S. populations. Mortality data were obtained from the Puerto Rico Central Cancer Registry and the Surveillance, Epidemiology, and End Results (SEER) program. Descriptive characteristics and temporal trends were derived via SEER*Stat software (version 9.0.42) and Joinpoint regression models, respectively. Relative survival was estimated using the Actuarial method and the Ederer II approach. Overall, CRC mortality trends showed a decline, but an increase in EOCRC mortality among Hispanics. PRH exhibited the lowest 5-year survival in regional cancers (54.10%), with NHB having the lowest survival among younger individuals. This study highlights significant disparities in EOCRC mortality trends and underscores an urgent need for targeted public health strategies and research efforts to address the disproportionate burden of EOCRC among PRH.

## 1. Introduction

Despite the overall decline in CRC incidence since the mid-1980s, primarily driven by the implementation of screening programs, there has been an alarming rise in early-onset (EOCRC) among those younger than 50 years old [1,2,3,4]. This increase has been accompanied by a rise in mortality rates in individuals under 55 years of age, which has shown an annual increase rate of 1% since the mid-2000s [1,4,5,6,7,8,9]. While the risk factors for EOCRC largely mirror those of overall CRC, including family history of colorectal neoplasia, predisposing germline mutations (e.g., Lynch syndrome), tobacco and alcohol use, physical inactivity, and obesity [10,11,12,13,14,15,16,17,18] the increasing incidence cannot be fully explained by known risk factors [19]. Moreover, cumulative evidence suggests that differences in early-life factors, including demographic and genetic variations, may contribute not only to disparities in EOCRC incidence but also significantly influence survival outcomes in younger patients [20].

Regional, racial, and ethnic disparities in CRC mortality and survival have previously been reported in the U.S. [1,21,22,23,24,25]. Non-Hispanic Blacks have the highest overall CRC incidence and the lowest survival rates, while U.S. Hispanics are more likely to present with advanced-stage disease and have worse survival rates compared to other populations [21]. However, grouping diverse populations, such as Hispanics, into a single category masks important differences in CRC incidence and mortality among subgroups [11]. For example, Cubans and Puerto Ricans in Florida had higher CRC mortality rates (18.9 and 19.9 per 100,000, respectively) than other USH subgroups, with rates ranging from 9.6–13.4 per 100,000 [5]. Racial/ethnic early-onset CRC disparities have also been reported, with NHB and USH having higher incidences compared to other population groups [6]. However, Hispanics have had the most marked increases, annually, in early-onset CRC incidence and the highest mortality [7,8,26].

Puerto Rico, a U.S. territory where 98.7% of the population identifies as Hispanic or Latino, regardless of race, had a disproportionate burden of CRC compared to populations living in the mainland U.S. [23,27,28]. Currently, CRC is the leading cause of cancer-related death on the island when combining men and women [22]. Despite this, very little is known regarding early-onset mortality and survival trends among PRH. This knowledge gap is particularly concerning given that Puerto Ricans are the second-largest Hispanic population in the U.S., with close to 5.6 million living in the mainland U.S. and 3.3 million on the island, making them central to understanding broader CRC patterns among U.S. Hispanics. In this updated analysis, we examine CRC mortality trends from 2000 to 2021 and assess survival rates for both early- and late-onset disease using data from the Puerto Rico Central Cancer Registry, which is not included in the Surveillance, Epidemiology, and End Results Program (SEER), and compare trends to those from other racial and ethnic groups from the mainland U.S. using data available from SEER. By comparing these trends to SEER data from mainland racial and ethnic groups, this study aims to fill a critical gap and inform targeted strategies for risk stratification, prevention, and treatment interventions among Puerto Ricans.

## 2. Materials and Methods

### 2.1. Data Sources and Study Population

CRC mortality data were obtained from two primary sources: the Puerto Rico Central Cancer Registry (PRCCR) and the U.S. Surveillance, Epidemiology, and End Results (SEER) program of the National Cancer Institute (NCI) from 1 January 2000 to 31 December 2021. Data from individuals aged 20 years or older were included in this analysis.

The PRCCR gathers cancer-related deaths data from the Puerto Rico Demographic Registry, and SEER from the National Center for Health Statistic. Both datasets follow the standards of the North American Association of Central Cancer Registries (NAACCR) and SEER, making data comparable for statistical purposes.

For survival analysis, the PRCCR incidence database and SEER-17 database were used. Only primary cases with a diagnostic and histologic confirmation of colorectal adenocarcinomas from 1 January 2012, to 31 December 2016, were included (ICD-O-3 site codes C18.0, C18.2–C18.9, C26.0, C19.9, and C20.9 and ICD-O-3 histology codes 8140, 8210–8211, 8255, 8261–8263, 8480–8481, 8490, and 8574).

### 2.2. Study Variables

The demographic variables examined included age at diagnosis (20–49 years vs. ≥50 years), gender (male vs. female), and racial/ethnic groups [Hispanics living in Puerto Rico (PRH), non-Hispanic Whites (NHW), non-Hispanic Blacks (NHB), non-Hispanic American Indians/Alaska Natives (NHAI/AN), non-Hispanic Asian or Pacific Islanders (NHAPI), U.S. mainland Hispanics (USH), and U.S. mainland overall].

### 2.3. Case Ascertainment

Mortality data from both PRCCR and SEER were collected for the specified timeframe. During this period, PR reported 14,371 CRC-related deaths, while SEER recorded 1,168,778 CRC-related deaths in the mainland U.S.

### 2.4. Statistical Analysis

Descriptive analyses were performed to summarize CRC-related deaths by race, ethnicity, age, and sex. Age-adjusted mortality rates (per 100,000 persons) were calculated using SEER*Stat software (version 9.0.42) and adjusted to the 2000 US Standard Population (CENSUS P25-1130). Chi-square (X^2^) tests were applied to examine associations between racial/ethnic groups, age groups (20–49 years vs. ≥50 years), and sex (male vs. female).

Trends in CRC mortality rates were analyzed using the Joinpoint Regression Program (version 5.1.0.0). This software identifies points where significant changes in trends occur, calculating Annual Percentage Change (APC) and 95% confidence intervals (CI) for each trend. The Average APC (AAPC), computed as a weighted average of the APCs, was used to summarize trends across the entire 2000–2021 study period.

The 1-, 3-, and 5-year relative survival rates were calculated using the PRCCR incidence case file and SEER-17 database. Relative survival, an alternative to calculating cancer-specific mortality, is calculated as the ratio of the observed survival to the expected survival for a group of people in a general population that is similar to that of the patient group with respect to race, sex, age, and calendar period of observation. For survival analyses, patients diagnosed between 2012 and 2016 were included, with follow-up through 2021. The relative survival of the PRH CRC subjects included in this study was calculated using the Puerto Rico lifetables and the strs command in Stata v.15.0. Relative survival for colorectal cancer was calculated stratified by sex, race/ethnicity, and age group, with further sub-analyses performed on the different stages. Survival rates were examined overall and with respect to the following demographic variables: age group (<50 and ≥50 years), and stage (localized, regional, and distant; unstaged was not included in the analysis).

## 3. Results

### 3.1. CRC Mortality by Racial and Ethnic Group

Descriptive analyses for a total of 2,349,560 CRC mortality cases by race/ethnicity during the 21-year period are presented in Table 1. When stratified by sex, CRC-related deaths were more frequent among men, accounting for the highest percentages of male deaths. USH had the highest percentage of CRC mortality cases among individuals younger than 50 years (12.2%), while PRH had the second highest among individuals aged 50 or older (93.8%), following NHW.

### 3.2. Age-Adjusted Average Annual Percent Changes (AAPC) by Racial and Ethnic Group

Analyses overall showed significantly declining mortality trends in all the racial and ethnic groups studied, irrespective of sex (Table 2), with marked decreases in women compared to men. Among those diagnosed with CRC age 50 or older (aver-age-onset CRC [AOCRC]), the lowest decreases in AAPCs among men and women were in NHAI/AN (AAPC_male_ = −1.36 *, 95% CI: −2.06 to −0.50 and AAPC_female_ = −1.01, 95% CI: −2.38 to 1.07). PRH showed the second lowest AOCRC decrease in men (AAPC_male_ = −1.54 *, 95% CI: −2.46 to −0.48); however, decreases in women were comparable to NHW and the U.S. overall. The highest significantly increasing EOCRC mortality trends were among PRH men and NHW women. Marked sex-based differences in EOCRC mortality trends were observed in PRH (AAPC_male_ = 2.77 *, 95% CI: 0.92–4.95 and AAPC_female_ = 0.14, 95% CI: −1.32–1.57), presenting a markedly higher mortality compared to USH (AAPC_male_ = 1.80 *, 95% CI: 1.02–2.47 and AAPC_female_ = 0.06, 95% CI: −0.57–1.13). NHW was the only group where females showed slightly higher EOCRC mortality compared to men. Among all groups, PRH had the second lowest decrease in EOCRC mortality trend during the study period, surpassed only by NHAI/AN populations (AAPC = 2.87, 95% CI: 1.30–4.78).

**Figure 1:**
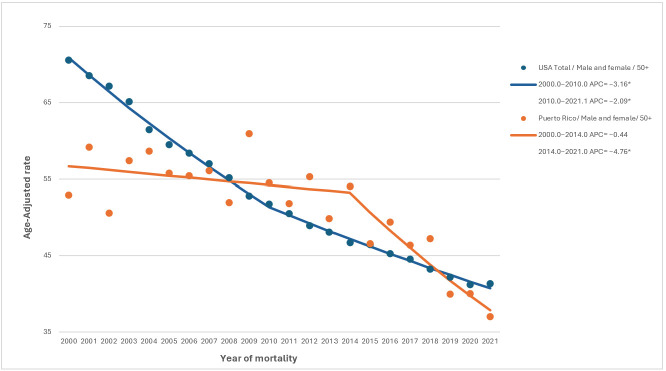
**Age-adjusted Annual Percent Change (AAPC) for AOCRC over 2000–2021 for PRH and United States (U.S.) overall.** PRH and overall US CRC mortality trends are highlighted in blue and orange, respectively. * Denotes significant change in temporal trend.

**Figure 2:**
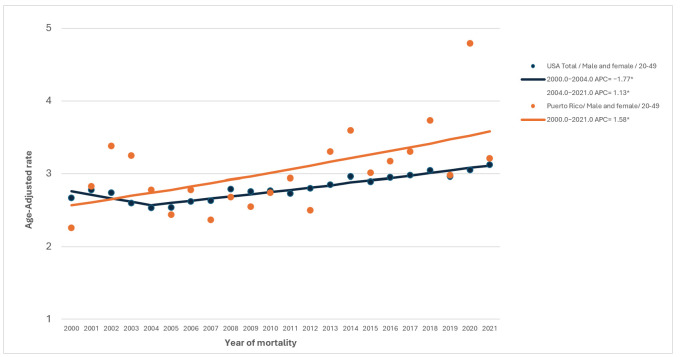
**Age-adjusted annual percent change (AAPC) for EOCRC during 2000–2021.** PRH and overall U.S. CRC mortality trends are highlighted in blue and orange, respectively. * Denotes significant change in temporal trend.

Joinpoint regression analyses revealed that PRH experienced a significantly more accelerated reduction in AOCRC mortality starting in 2014, with an APC of −4.76 (Figure 1). However, a consistently increasing EOCRC mortality trend was observed among PRH since 2000 (AAPC = 1.58, 95% CI 0.41 to 2.83), whereas a decrease from 2000 to 2004; and an increase beginning in 2005 was observed in the overall U.S. population (Figure 2).

### 3.3. Disparities in Relative Survival by Age and Stage at Diagnosis

Cumulative 5-year relative survival outcomes for CRC according to age and stage at diagnosis are presented in Table 3. Among EOCRC patients, NHW had the highest CRC survival (69.5%) and NHB the lowest (58.9%), whereas the remaining racial/ethnic groups showed comparable rates within this age group. Similarly, NHB had the lowest survival (58.8%), followed by PRH (62.6%), among those diagnosed with AOCRC. Stage-specific analyses showed that PRH had the lowest 5-year relative survival in localized and regional cancers (85.5% and 61.1%, respectively); NHB and PRH had the lowest survival in distant disease.

### 3.4. Relative Survival Analysis by Sex

Table 4 presents observed survival outcomes for 1-, 3-, and 5-years after EOCRC diagnosis according to sex and race/ethnicity between 2012 and 2016. Overall, females showed higher survival than males at each time point. Analysis showed that NHB has consistently lower 1-, 3-, and 5-year relative survival rates in men and women, except for 1-year relative survival in NHAI/AN women, who had the lowest rate. Among men, PRH had the second lowest EOCRC relative survival rates at all time points, with comparable 5-year rates to USH. Among women, and when combining both sexes, PRH had the lowest 1-year relative survival.

## 4. Discussion

Our study provides the first comprehensive mortality and survival rates for Hispanics living in Puerto Rico compared to updated data from other U.S. populations. According to the 2020 US Census, Hispanics comprise 18.7% of the U.S. population [29], with Puerto Ricans accounting for 9% of this group [30]. Given that colorectal cancer is the leading cause of cancer-related death among both men and women in Puerto Rico [22], Puerto Ricans living on the U.S. mainland likely contribute significantly to the colorectal cancer trends observed among U.S. Hispanics, highlighting the need to study this specific subpopulation.

### 4.1. Overall and Average-Onset CRC Trends

When analyzing mortality trends from 2000 to 2021, a significant decline was observed in both overall and late-onset CRC across racial and ethnic groups, consistent with findings from previous studies [27,31]. However, greater year-to-year variability was observed for the Puerto Rico cohort compared with the US mainland, likely reflecting fluctuations associated with smaller population size and annual case counts rather than true instability in mortality trends. Hispanic populations, both in Puerto Rico and on the U.S. mainland, had markedly smaller decreases in mortality than the overall U.S. population. The comparable decreasing mortality trends between these two groups may reflect shared cultural and behavioral factors, as well as barriers to access to healthcare [31]. Systemic factors, such as uneven distribution of oncology services, fragmentation of care, and insurance-related barriers, may also contribute to the mortality trends observed among Hispanics [31,32]. Among PRH, a significant shift in mortality trends was observed starting in 2014, which may be associated with gradual improvements in cancer treatment capacity and policy reforms, such as the implementation of Law No. 107-2012, which ensured equal insurance coverage for all chemotherapy modalities, including intravenous, oral, injectable, and intrathecal treatments [33]. This lehislation likely enhanced treatment accessibility and continuity, potentially contributing to the decline in mortality observed in subsequent years.

Survival analysis for CRC during 2012–2016 showed that PRH had the lowest 5-year survival among adults aged 50 and older, following NHB. This is consistent with a previous publication from our group [23], showing that this trend persists. However, NHB have been reported to have a higher percentage of cases diagnosed at advanced stages compared to PRH, suggesting that, in addition to biological differences, other factors beyond stage at diagnosis may contribute to worse outcomes among PRH [24]. Also, close to half of the population in Puerto Rico has government health plans (e.g., Medicaid/Medicare) [34]. Previous studies have reported that patients insured under government health plan exhibit significantly lower CRC survival compared with those covered by private insurace [35], suggesting that delays in treatment initiation and limitations within the public insurance sector may contribute to the observed survival disparities among PRH.

### 4.2. Early-Onset CRC Trends

Significant increases in EOCRC mortality were observed across all racial and ethnic groups, with PRH having the second-highest EOCRC mortality trend. These findings contrast with the overall decline in CRC mortality, suggesting a shifting burden of disease toward younger populations. The disparate increase in EOCRC mortality observed among PRH compared with USH and the general U.S. population may reflect unequal access to timely and effective treatment, lower access to health education, and socioeconomic barriers across the island [36,37,38,39]. These systemic limitations may contribute to delayed treatment initiation and higher mortality in Puerto Rico, where younger patients often rely on public insurance and face longer waiting times for oncology services.

In contrast, PRH had higher survival rates compared to USH, consistent with previous studies [22,23,40]. One important consideration is that the Hispanic population in the mainland United States is highly heterogeneous, comprising individuals from diverse countries of origin with wide variations in genetic ancestry, socioeconomic status, healthcare access, cultural practices, and cancer risk profiles. As a result, this aggregate data on “Hispanics” in the mainland may obscure important differences among subgroups, including those that affect CRC outcomes. PRH represents a more genetically and culturally homogeneous group, with a shared healthcare system. Puerto Ricans have a distinct admixture of European, African, and Indigenous populations [41], which may influence both CRC risk and outcomes differently than other Hispanic subgroups. Nevertheless, despite their higher EOCRC survival rates compared to USH, 1- and 3-year survival among PRH remains lower than that of the overall U.S. population, highlighting the need for further investigation into the factors contributing to this persistent disparity.

### 4.3. Key Contributor to CRC Disparities in PRH

Puerto Rico, as a U.S. territory with distinctive environmental and social conditions, faces a particularly vulnerable scenario regarding CRC mortality. Previous research suggests that modifiable risk factors, such as socioeconomic status, diet, and access to healthcare, intersect with non-modifiable factors, including genetic predisposition and ancestry composition, to shape clinical outcomes [5,22,42]. Lower screening adherence, limited availability of advanced therapies, and unequal quality of care have been identified as major contributors to the higher mortality rates observed among PRH compared to other populations [35,43]. Moreover, evidence from local studies indicates that delays in diagnostic evaluation and initiation of treatment remain common [24,31], particularly among patients receiving care in the public sector, contributing to poorer outcomes despite improvements in coverage and treatment availability [35]. Collectively, these factors illustrate how clinical and structural limitations continue to drive CRC mortality and survival disparities in Puerto Rico.

### 4.4. Future Research

The present study had notable strengths, including its ability to distinguish PRH residing on the island from the broader U.S. Hispanic population, offering a clearer understanding of population-specific trends often lacking in large-scale epidemiological studies. While full disaggregation of Hispanic subgroups in the U.S. was not possible due to limitations in SEER coding, the inclusion of island-based PRH offers unique insights into a subgroup that is not included in SEER data, with distinct environmental, socioeconomic, and structural exposures. However, the study was limited by the lack of geographic information for cases in Puerto Rico, which restricted evaluation of regional disparities in access to care, as most major hospitals are located in the metropolitan areas on the island. Another limitation is the inability to disaggregate the USH population in SEER by ancestral or country of origin. Therefore, comparisons with PRH should be interpreted cautiously. Additionally, incomplete data on tumor stage may have affected survival analyses and reduced the precision of stratified comparisons.

Future research should prioritize the generation and disaggregation of population-specific data to better tailor personalized screening and prevention strategies that account for local barriers, biological diversity, and ancestry-related factors. Strengthening molecular characterization and integrating these findings with environmental and social determinants will deepen our understanding of the biological and structural factors driving CRC disparities in Puerto Rico. Strengthening local research efforts will be essential to designing interventions that address the specific needs of this Hispanic subpopulation, especially as the continuous exodus of Puerto Ricans to the U.S. mainland has significantly reshaped the island’s demographic landscape and impacted CRC epidemiological trends. Although migration from Puerto Rico to the mainland may influence population health characteristics, this movement often includes individuals seeking specialized care or improved access to medical services, which may attenuate the traditional “healthy migrant” effect described in other populations. Understanding how CRC affects those who remain on the island is vital for guiding public health planning both in Puerto Rico and the mainland. Developing tailored, precision-based prevention and targeted therapies will be essential to increase early detection and improve outcomes.

## 5. Conclusions

In sum, this presents a comprehensive analysis of CRC mortality and survival among PRH compared to other U.S. populations, revealing persistent disparities driven by a combination of biological, social, and structural factors. Although overall CRC mortality has declined across racial and ethnic groups, reductions among Hispanics on the island and the mainland have been less pronounced. Moreover, PRH had particularly low survival rates in regional cancers and rising EOCRC mortality trends. Moving forward, targeted research efforts focused on generating population-specific, disaggregated data and integrating molecular, environmental, and social determinants will be essential to develop precision-based prevention, early detection, and treatment strategies tailored to Hispanic subpopulations. Such efforts are critical to improve CRC outcomes and guide public health interventions both on the island and in the mainland U.S.

## Figures and Tables

**Table 1:** Descriptive statistics on CRC deaths per race and ethnicity group (2000–2021).

Characteristics	CRC Mortality							X^2^ (Degrees of Freedom)
	PRH	NHW	NHB	NHAI/AN	NHAPI	USH	U.S.	
**Total cases**	14,371	905,347	151,133	6185	31,746	72,000	1,168,778	
Sex, *n* (%)								
Male	7949 (55.3)	466,099 (51.5)	76,907 (50.9)	3348 (54.1)	16,500 (52.0)	40,101 (55.7)	604,324 (51.7)	607.78 * (6)
Female	6422 (44.7)	439,248 (48.5)	74,226 (49.1)	2837 (45.9)	15,246 (48.0)	31,899 (44.3)	564,454 (48.3)	
Age at death, *n* (%)								
20–49	885 (6.2)	48,857 (5.4)	14,342 (9.5)	652 (10.5)	3409 (10.7)	8794 (12.2)	76,207 (6.5)	9.0 × 10^3^ * (6)
≥50	13,486 (93.8)	856,490 (94.6)	136,791 (90.5)	5533 (89.5)	28,337 (89.3)	63,206 (87.8)	1,092,571 (93.5)	

Hispanics living in Puerto Rico (PRH), Non-Hispanic Whites (NHW), Non-Hispanic Blacks (NHB), U.S. Non-Hispanic Asian or Pacific Islanders (NHAPI), U.S. Non-Hispanic American Indians/Alaska Natives (NHAI/AN), U.S. Hispanics (USH), U.S. Overall (U.S.); * The X^2^ value is significantly different from zero at the alpha = 0.05 level.

**Table 2:** Age-adjusted Annual Average Percent Change (AAPC) for CRC mortality per race and ethnicity group from 2000–2021.

Characteristics		CRC Age-Adjusted Mortality AAPC (95% CI)						
		PRH	NHW	NHB	NHAI/AN	NHAPI	USH	U.S.
Male	20–49 yo	2.77 * (0.92 to 4.95)	1.15 * (0.86 to 1.61)	−0.79 * (−1.26 to −0.12)	**	0.86 * (0.03 to 1.84)	1.80 * (1.02 to 2.47)	0.63 * (0.38 to 1.08)
	≥50 yo	−1.54 * (−2.46 to −0.48)	−2.68 * (−2.77 to −2.59)	−2.69 * (−2.84 to −2.54)	−1.36 * (−2.06 to −0.50)	−2.17 * (−2.43 to −1.85)	−1.72 * (−1.88 to −1.52)	−2.65 * (−2.73 to −2.58)
	Overall (≥20 yo)	−1.33 * (−2.27 to −0.23)	−2.38 * (−2.44 to −2.31)	−2.52 * (−2.67 to −2.35)	−0.097 * (−1.56 to −0.25)	−1.92 * (−2.18 to −1.60)	−1.51 * (−1.65 to −1.33)	−2.39 * (−2.46 to −2.31)
Female	20–49 yo	0.14 (−1.32 to 1.57)	1.24 * (0.83 to 1.66)	−0.99 * (−1.46 to −0.52)	**	−0.04 (−0.88 to 0.97)	0.06 (−0.57 to 1.13)	0.47 * (0.19 to 0.87)
	≥50 yo	−2.17 * (−2.90 to −1.48)	−2.55 * (−2.65 to −2.45)	−3.21 * (−3.43 to −2.98)	−1.01 (−2.38 to 1.07)	−1.94 * (−2.44 to −1.32)	−1.83 * (−2.17 to −1.58)	−2.66 * (−2.79 to −2.53)
	Overall (≥20 yo)	−1.87 * (−2.82 to −0.79)	−2.25 * (−2.34 to −2.15)	−3.01 * (−3.21 to −2.81)	−0.73 (−2.16 to 1.45)	−1.76 * (−2.23 to −1.17)	−1.61 * (−1.89 to −1.36)	−2.39 * (−2.51 to −2.28)
Overall	20–49 yo	1.58 * (0.41 to 2.83)	1.16 * (0.85 to 1.58)	−0.93 * (−1.30 to −0.38)	2.87 * (1.30 to 4.78)	0.77 (−0.03 to 1.57)	1.53 * (0.89 to 2.31)	0.57 * (0.38 to 0.81)
	≥50 yo	−1.90 * (−2.52 to −1.29)	−2.55 * (−2.62 to −2.47)	−2.89 * (−3.02 to −2.75)	−1.28 * (−2.08 to −0.07)	−2.01 * (−2.28 to −1.67)	−1.77 * (−1.91 to −1.59)	−2.61 * (−2.71 to −2.51)
	Overall (≥20 yo)	−1.56 * (−2.39 to −0.70)	−2.27 * (−2.35 to −2.19)	−2.70 * (−2.82 to −2.58)	−0.92 (−1.75 to 0.45)	−1.79 * (−2.06 to −1.46)	−1.52 * (−1.67 to −1.33)	−2.35 * (−2.43 to −2.25)

Hispanics living in Puerto Rico (PRH), Non-Hispanic Whites (NHW), Non-Hispanic Blacks (NHB), U.S. Non-Hispanic Asian or Pacific Islanders (NHAPI), U.S. Non-Hispanic American Indians/Alaska Natives (NHAI/AN), U.S. Hispanics (USH), United States Overall (U.S.); * The age-adjusted AAPC is significantly different from zero at an alpha ≤ 0.05; ** At least one of the years do not have enough cases to calculate the AAPC.

**Table 3:** 5-year relative survival for CRC by race/ethnicity according to age and tumor stage at diagnosis of incident CRC during 2012–2016 with follow-up through 2021.

Characteristics	PRH (n = 6996) % Survival (CI)	NHW (n = 80,511) % Survival (CI)	NHB (n = 14,101) % Survival (CI)	NHAI/AN (n = 1071) % Survival (CI)	NHAPI (n = 11,846) % Survival (CI)	USH (n = 16,463) % Survival (CI)
Age group						
20–49 yo	64.6 (60.8–68.1)	69.5 (68.5–70.5)	58.9 (56.6–61.1)	65.1 (56.9–72.2)	68.0 (65.5–70.3)	63.4 (61.5–65.2)
≥50 yo	62.6 (61.2–64.0)	66.8 (66.3–67.2)	58.8 (57.7–59.8)	65.5 (61.5–69.1)	67.5 (66.4–68.5)	64.9 (63.9–65.8)
Stage at Diagnosis						
Localized	85.5 (83.7–87.2)	91.0 (90.4–91.5)	87.0 (85.5–88.3)	91.4 (85.5–95.0)	89.9 (88.6–91.1)	88.8 (87.6–89.9)
Regional	61.1 (59.0–63.2)	73.6 (73.0–74.2)	69.4 (67.9–70.9)	71.3 (65.4–76.4)	74.7 (73.2–76.1)	72.7 (71.4–73.9)
Distant	11.8 (9.9–13.9)	15.4 (14.8–16.0)	11.3 (10.3–12.4)	16.1 (11.6–21.3)	17.0 (15.4–18.6)	15.7 (14.5–17.0)

PR Hispanics (PRH), Non-Hispanic Whites (NHW), Non-Hispanic Blacks (NHB), U.S. Non-Hispanic Asian or Pacific Islanders (NHAPI), U.S. Non-Hispanic American Indians/Alaska Natives (NHAI/AN), US Hispanics (USH).

**Table 4:** 1-, 3- and 5-year relative survival by sex and race/ethnicity for individuals younger than 50 years diagnosed with CRC during 2012–2016 with follow-up through 2021.

	Male % Survival (CI)			Female % Survival (CI)			Overall % Survival (CI)		
	1-year	3-year	5-year	1-year	3-year	5-year	1-year	3-year	5-year
PRH	85.5 (81.2–88.8)	66.8 (61.5–71.6)	61.0 (55.5–66.1)	88.5 (84.5–91.5)	75.6 (70.5–79.9)	68.3 (62.9–73.1)	86.9 (84.1–89.3)	71.1 (67.5–74.4)	64.6 (60.8–68.1)
NHW	91.3 (90.4–92.1)	76.2 (74.9–77.4)	68.2 (66.8–69.5)	92.7 (91.8–93.5)	78.9 (77.6–80.2)	71.1 (69.6–72.6)	91.9 (91.3–92.5)	77.4 (76.5–78.3)	69.5 (68.5–70.5)
NHB	87.3 (84.9–89.3)	64.9 (61.7–67.9)	54.3 (50.9–57.5)	90.0 (88.0–91.7)	70.8 (67.8–73.6)	63.2 (60.1–66.2)	88.7 (87.2–90.0)	67.9 (65.8–70.0)	58.9 (56.6–61.1)
NHAI/AN	91.2 (82.4–95.7)	74.7 (63.4–82.9)	63.7 (51.8–73.3)	83.7 (73.3–90.3)	73.9 (62.3–82.5)	66.7 (54.5–76.3)	87.7 (81.4–91.9)	74.3 (66.6–80.6)	65.1 (56.9–72.2)
NHAPI	91.6 (89.5–93.3)	75.1 (72.0–78.0)	64.9 (61.5–68.1)	93.6 (91.6–95.2)	79.1 (75.9–81.9)	71.5 (68.0–74.7)	92.5 (91.1–93.7)	77.0 (74.8–79.0)	68.0 (65.5–70.3)
USH	91.0 (89.4–92.3)	71.8 (69.4–74.1)	60.9 (58.3–63.4)	92.9 (91.4–94.2)	75.2 (72.8–77.5)	66.3 (63.5–68.8)	91.9 (90.8–92.8)	73.4 (71.7–75.0)	63.4 (61.5–65.2)

Hispanics living in Puerto Rico (PRH), Non-Hispanic Whites (NHW), Non-Hispanic Blacks (NHB), U.S. Non-Hispanic Asian or Pacific Islanders (NHAPI), U.S. Non-Hispanic American Indians/Alaska Natives (NHAI/AN), U.S. Hispanics (USH).

## Data Availability

The data analyzed in this study is available upon request through the Puerto Rico Central Cancer Registry and the Surveillance, Epidemiology, and End Results (SEER) Program.

## Abbreviations

The following abbreviations are used in this manuscript:

CRC	Colorectal Cancer
EOCRC	Early-Onset Colorectal Cancer
U.S.	United States
PR	Puerto Rico
USH	Hispanics living in the United States
PRH	Hispanics residing in Puerto Rico
NHW	Non-Hispanic Whites
NHB	Non-Hispanic Blacks
NHAPI	Non-Hispanic Asian or Pacific Islanders
NHAI/AN	U.S. Non-Hispanic American Indians/Alaska Natives
SEER	Puerto Rico Central Cancer Registry and the Surveillance, Epidemiology, and End Results
PRCCR	Puerto Rico Central Cancer Registry
AAPC	Average Annual Percent Change
NCI	National Cancer Institute
NAACCR	North American Association of Central Cancer Registries
CI	Confidence Intervals
